# The Need for Personalized Approaches to Microbiome Modulation

**DOI:** 10.3389/fpubh.2020.00144

**Published:** 2020-04-29

**Authors:** Nita Jain

**Affiliations:** Independent Researcher, Lilburn, GA, United States

**Keywords:** microbiome, personalized medicine, gut ecology, metagenomics, FMT, host-microbe interactions

## Introduction

The human microbiome has been a topic of interest for both research and clinical applications in recent decades. However, the considerable gut microbial variation observed across human populations poses a challenge in terms of targeted interventions. Diet (1), exercise (2), age (3), ancestry (4), and geographic latitude (5) all influence the composition of the gut microbiome. The individualized nature of microbiome compositions makes responses to modulatory interventions, including probiotics, prebiotics, and fecal microbiota transplantation, subject-specific (6).

## Microbiome Variability and Host Characteristics

### Host Characteristics Influence Individual Variation in Gut Microbiota Composition

Host features govern the types of niches available for occupation such that only microbes adapted to host ecological conditions can successfully colonize. For this reason, autochthonous strains are more likely to possess the traits necessary to successfully persist in gut ecosystems, accounting for the failure of most allochthonous probiotic strains to colonize. Genes that encode for traits such as mucosal adherence and acid resistance can confer greater ecological fitness in a host environment (6). Habitat filters are influenced by various factors, including a host's genetics, metabolism, diet, and environment, and select for microbes with common traits, leading to phylogenetic underdispersion. Out of the hundreds of phyla encountered in terrestrial and aquatic ecosystems, the human gut is dominated by only five, illustrating the impact of selection through habitat filters (7). Certain gene polymorphisms can differentially impact intestinal microbiota composition through provision of adhesion sites and growth substrates such as secreted glycans (6).

The *FUT2* gene encodes for fucosyltransferase, which is responsible for the synthesis of the H antigen that serves as the precursor to the ABH histo-blood group antigens in mucus and other bodily secretions. Individuals that are homozygous for any non-functional *FUT2* allele are known as non-secretors and will not present ABH antigens on epithelial cell surfaces whereas individuals carrying at least one functional *FUT2* allele will express ABH antigens on intestinal mucosal surfaces (8). Secretor status determines the expression of fucosylated glycan epitopes in the human intestine, and the *FUT2* non-secretor phenotype has been linked to alterations in the gut microbiome in the form of reduced bifidobacterial diversity, richness, and abundance (9). However, large-scale studies have not been able to replicate these reports (10, 11). Collecting and analyzing additional metadata on diet and lifestyle habits may help resolve some of the discrepancies observed between different studies. A murine study, for example, showed that *FUT2* secretor status-associated changes in intestinal microbiota composition are diet-dependent (12). Citizen science initiatives, such as the American Gut Project, can help evaluate the effects of interactions among host genetics, diet, and environment on microbiome composition on larger scales. Analyzing patient sequencing data along with self-reported metadata may also help elucidate possible associations between the non-secretor phenotype and increased risk of certain diseases, including Crohn's disease, type 1 diabetes, vaginal candidiasis, and urinary tract infections (13).

### Efficacy of Microbiome Modulatory Interventions Depend on Baseline Host Characteristics

An individual's baseline microbiota composition determines the types of dietary fibers that may be fermented to produce short-chain fatty acids (SCFAs) such as butyrate. The microbiomes of some individuals may be capable of fermenting pectin to produce SCFAs while the microbiomes of others may require inulin to achieve the same effect (14). The inherent heterogeneity among the gut microbiota of healthy humans differentially affects functional degradation of fibers and the SCFAs produced in response (15). Dietary fiber may also improve glucose homeostasis in a subset of patients through colonic production of SCFAs; acetate and butyrate have been shown to stimulate production of glucagon-like peptide-1 (GLP-1) and peptide YY (PYY), which in turn stimulate insulin secretion. A study examining dietary fiber interventions in type 2 diabetes patients found that the microbiomes of positive responders possessed more genes for plant fiber utilization while the microbiomes of negative responders were more enriched in genes for utilization of animal carbohydrates derived from mucin (16). Data on a patient's microbiome composition may therefore be able to inform personalized dietary intervention strategies targeted toward increased SCFA production in order to ameliorate disease phenotypes.

Before targeting colonic SCFA production, it may be judicious to first evaluate a patient's immune system activity in order to prevent potential adverse effects. A study conducted by a research group from the Massachusetts Institute of Technology used microphysiological systems to demonstrate that SCFAs can either ameliorate or exacerbate ulcerative colitis disease severity depending on the activation state of CD4 T cells. In the setting of T cell-mediated acute inflammation, SCFAs led to further gut barrier disruption and hepatobiliary damage (17). Observations from a randomized controlled trial (RCT) on the use of fecal microbiota transplantation (FMT) in ulcerative colitis patients further elucidate the interface between immune system activation and host response to the resident gut microbiota, as individuals on immunosuppressive therapy were more likely to benefit from FMT compared to patients who were not on immunosuppressive therapy (46 vs. 15%) (18). Thus, immune-modulating strategies may theoretically help facilitate successful strain engraftment.

### Individualized Microbiome Features Govern Strain Engraftment Efficacy

Exogenous species are more likely to successfully engraft through FMT when related species are already present (19). In light of this knowledge, reducing recipient microbial load with antibiotics may hinder successful engraftment of the donor microbiota (20). A study evaluating the effects of Rifaximin pre-treatment compared to FMT alone for the treatment of ulcerative colitis reported no significant difference between groups in terms of disease activity (21). Changes to gut microbiota composition caused by colonic lavage or laxative use may also have unintended effects on FMT efficacy (22). While Li et al. reported that new strains transfer more easily than new species, Stecher et al. similarly described a “like will to like” principle, suggesting that successful colonization of both pathogenic and commensal strains is dictated by prior establishment of related species (23). A research group from the University of Milan observed a significant increase in Proteobacteria abundance and a significant decrease in Firmicutes abundance at the phylum level immediately after colon cleansing (24). Considering the implication of high Proteobacteria abundance in various human diseases (25) and its possible utility as a marker for dysbiosis (26), bowel preparation procedures prior to fecal transplantation may negatively impact FMT efficacy by preferentially facilitating the engraftment of potential pathobionts, but this possibility would warrant further research.

While conspecific strains exhibit greater colonization success than new species (19), ecology theory conversely predicts that competition among phylogenetically related strains will be greater as a result of trait similarity and niche overlap. Consequently, the presence of certain strains at baseline may prevent the colonization of other strains within the same species due to competitive exclusion, or phylogenetic limiting. A study examining strain engraftment in the human gut found that *B. longum* subsp. *longum* AH1206 was more likely to successfully engraft in hosts who did not already harbor native *B. longum* strains, suggesting niche availability as a limiting factor for persistence. However, while baseline *B. longum* abundance generally inversely correlated with AH1206 persistence, this pattern did not hold true for all subjects. Since traits that define niches are not always phylogenetically conserved within species, researchers also evaluated metagenomic data in order to assess differences in functional microbiome composition between persisters and non-persisters. Specifically, AH1206 was able to engraft in a subset of subjects whose microbiome lacked certain carbohydrate utilization genes characteristic of *B. longum* strains (27). In regards to niche availability, functional gene distinctions may be more predictive of exclusion effects than phylogenetic considerations under certain environmental conditions, as horizontal gene transfer can facilitate the emergence of functionally similar bacteria in phylogenetically distinct taxa (28). The factors that determine whether habitat filtering or competitive exclusion takes precedence will likely include contextual and taxonomic considerations (6).

### Considerations of Host-Microbe Coevolution May Enhance Efficacy of FMT

Whereas sharing a joint evolutionary history is a characteristic of autochthony (6), colonization of strains that did not evolve with a given host may result in hologenomic disequilibrium and cause negative health effects in the form of certain increased disease risks. For example, the presence of a specific strain of *Helicobacter pylori* in a host that did not coevolve with that microorganism was associated with an increased risk of gastric cancer (29). Additionally, the equilibrium of hunter-gatherer microbiota may be disrupted after exposure to a Western diet and switch to a state of dysbiosis. Thus, classifications of “commensal” and “pathogenic” may be relative and dependent on evolutionary history among other considerations, underlying the importance of stratifying stool donors by ethnogeographic and social factors (30). Furthermore, most metagenomic studies sample both study and control groups from the same population, which is exposed to similar environmental conditions common to urban lifestyles in developed countries, suggesting that many “healthy” subjects may simply be in a prodromal period. For these reasons, FMT donor screening on the basis of pathogen testing alone may be insufficient to prevent potential adverse outcomes in recipients, and more rigorous screening may include clinical laboratory data as well as metagenomic analyses.

### Clinical Outcomes in Response to FMT May Be Donor-Dependent

Microbial communities that exhibit greater phylogenetic diversity and evenness are considered more resilient to invasion. As a result, fecal microbiota transplantation results in a higher degree of engraftment in patients exhibiting severe microbiome perturbation, such as that encountered in the setting of active *C. difficile* infection, compared to patients with metabolic syndrome (19). High levels of genetic diversity in an incoming community increase the chance of successful invasions, as some organisms will likely possess the adaptations necessary to thrive. In particular, high microbial richness has been shown to be one of the most important factors in determining FMT outcome (31). While most literature on FMT has focused on bacteria as the therapeutically active agent, recent research suggests that phages may play a more significant role in disease resolution than previously realized. Donor-derived phages may target indigenous species of the host microbiome, expanding niche availability for incoming microbes. Zuo et al. reported that treatment response to FMT was associated with bacteriophage transfer involving *Caudovirales* taxa (32).

Based on the condition being treated, donors with certain microbiota profiles may be more effective than others. For example, fecal microbiota enriched with *Bifidobacterium* has been shown to be a positive predictor for the efficacy of FMT in IBS patients. Donor material rich in *Bifidobacterium* may stimulate the growth and expansion of undetectable strains in recipient microbiota to match the level of diversity observed in donor microbiota (33). In this manner, the efficacy of FMT likely depends upon stimulation of recipient microbiota by donor material rather than literal “transplantation” of donor microbiota. Similarly, an RCT examining the effects of FMT in patients with ulcerative colitis found that remission among responders was associated with increases in bacterial abundances of *Clostridium* clusters IV and XIVa (34). Another RCT involving UC patients found that FMT treatment success in response to one particular donor, donor B, was 39% vs. 10% for other donors, providing further evidence that clinical outcomes may be donor-dependent. The two most commonly used donors in the study, donor A and donor B, displayed significant differences in taxonomic composition, including enrichment in the family Lachnospiraceae and the genus *Ruminococcus* in donor B and enrichment in the order Clostridiales and the genera *Escherichia* and *Streptococcus* in donor A (18). The donor-dependent nature of FMT efficacy may help explain the disparity in clinical results observed among different studies conducted on a specific condition.

## Conclusion

Given the considerable amount of variation observed in human populations, bridging the gap between microbiome research and clinical applications may allow for more targeted, personalized recommendations based on diet, ancestry, geography, and physiology as well as microbial phylogenetic, metagenomic, and metabolic considerations.

## Author Contributions

The author confirms being the sole contributor of this work and has approved it for publication.

## Conflict of Interest

The author declares that the research was conducted in the absence of any commercial or financial relationships that could be construed as a potential conflict of interest.

## References

[1] David LA Maurice CF Carmody RN Gootenberg DB Button JE Wolfe BE. Diet rapidly and reproducibly alters the human gut microbiome. Nature. (2014) 505:559–63. 10.1038/nature12820 24336217 PMC3957428

[2] Grosicki GJ Durk RP Bagley JR. Rapid gut microbiome changes in a world-class ultramarathon runner. Physiol Rep. (2019) 7:e14313. 10.14814/phy2.14313 31872558 PMC6928244

[3] Salazar N Arboleya S Fernández-Navarro T de Los Reyes-Gavilán CG Gonzalez S Gueimonde M. Age-associated changes in gut microbiota and dietary components related with the immune system in adulthood and old age: a cross-sectional study. Nutrients. (2019) 11:1765. 10.3390/nu11081765 31370376 PMC6722604

[4] Goodrich JK Waters JL Poole AC Sutter JL Koren O Blekhman R. Human genetics shape the gut microbiome. Cell. (2014) 159:789–99. 10.1016/j.cell.2014.09.053 25417156 PMC4255478

[5] Suzuki TA Worobey M. Geographical variation of human gut microbial composition. Biol Lett. (2014) 10:20131037. 10.1098/rsbl.2013.1037 24522631 PMC3949373

[6] Walter J Maldonado-Gómez MX Martínez I. To engraft or not to engraft: an ecological framework for gut microbiome modulation with live microbes. Curr Opin Biotechnol. (2018) 49:129–39. 10.1016/j.copbio.2017.08.008 28866242 PMC5808858

[7] Catford JA Jansson R Nilsson C Reducing redundancy in invasion ecology by integrating hypotheses into a single theoretical framework. Divers Distribut. (2009) 15:22–40. 10.1111/j.1472-4642.2008.00521.x

[8] Ferrer-Admetlla A Sikora M Laayouni H Esteve A Roubinet F Blancher A. A natural history of FUT2 polymorphism in humans. Mol Biol Evol. (2009) 26:1993–2003. 10.1093/molbev/msp108 19487333

[9] Wacklin P Mäkivuokko H Alakulppi N Nikkil,ä J Tenkanen H Räbinä J. Secretor genotype (FUT2 gene) is strongly associated with the composition of Bifidobacteria in the human intestine. PLoS ONE. (2011) 6:e20113. 10.1371/journal.pone.0020113 21625510 PMC3098274

[10] Davenport ER Goodrich JK Bell JT Spector TD Ley RE Clark AG ABO antigen and secretor statuses are not associated with gut microbiota composition in 1,500 twins. BMC Genomics. (2016) 17:941 10.1186/s12864-016-3290-1 27871240 PMC5117602

[11] Turpin W Bedrani L Espin-Garcia O Xu W Silverberg MS Smith MI FUT2 genotype and secretory status are not associated with fecal microbial composition and inferred function in healthy subjects. Gut Microbes. (2018) 9:357–68. 10.1080/19490976.2018.1445956 29533703 PMC6219652

[12] Kashyap PC Marcobal A Ursell LK Smits SA Sonnenburg ED Costello EK. Genetically dictated change in host mucus carbohydrate landscape exerts a diet-dependent effect on the gut microbiota. Proc Natl Acad Sci USA. (2013) 110:17059–64. 10.1073/pnas.1306070110 24062455 PMC3800993

[13] Wacklin P Tuimala J Nikkilä J Sebastian T Mäkivuokko H Alakulppi N. Faecal microbiota composition in adults is associated with the FUT2 gene determining the secretor status. PLoS ONE. (2014) 9:e94863. 10.1371/journal.pone.0094863 24733310 PMC3986271

[14] Gurry T HST Microbiome Consortium Gibbons SM Nguyen LTT Kearney SM Ananthakrishnan A. Predictability and persistence of prebiotic dietary supplementation in a healthy human cohort. Sci Rep. (2018) 8:12699. 10.1038/s41598-018-30783-1 30139999 PMC6107591

[15] Gurry T Nguyen LTT Yu X Alm EJ Functional heterogeneity in the fermentation capabilities of the healthy human gut microbiota. bioRxiv [preprint]. (2020). 10.1101/2020.01.17.910638 PMC8294568 34288919

[16] Zhao L Zhang F Ding X Wu G Lam YY Wang X. Gut bacteria selectively promoted by dietary fibers alleviate type 2 diabetes. Science. (2018) 359:1151–56. 10.1126/science.aao5774 29590046

[17] Trapecar M Communal C Velazquez J Maass CA Huang YJ. Gut-Liver physiomimetics reveal paradoxical modulation of IBD-related inflammation by short-chain fatty acids. bioRxiv [preprint]. (2019). 10.1101/706812 32191873 PMC8143761

[18] Moayyedi P Surette MG Kim PT Libertucci J Wolfe M Onischi C. Fecal microbiota transplantation induces remission in patients with active ulcerative colitis in a randomized controlled trial. Gastroenterology. (2015) 149:102–9.e6. 10.1053/j.gastro.2015.04.001 25857665

[19] Li SS Zhu A Benes V Costea PI Hercog R Hildebrand F. Durable coexistence of donor and recipient strains after fecal microbiota transplantation. Science. (2016) 352:586–9. 10.1126/science.aad8852 27126044

[20] Manichanh C Reeder J Gibert P Varela E Llopis M Antolin M. Reshaping the gut microbiome with bacterial transplantation and antibiotic intake. Genome Res. (2010) 20:1411–9. 10.1101/gr.107987.110 20736229 PMC2945190

[21] El-Nachef N Kassam Z Piceno YM Ablaza A.-J. Zydek M Does Rifaximin prior to fecal microbiota transplantation improve clinical outcomes compared to microbiome restoration alone in ulcerative colitis? A cohort study evaluating the impact of non-absorbable antibiotic pretreatment. Gastroenterology. (2017) 152:S1008–9. 10.1016/S0016-5085(17)33421-2

[22] Tropini C Moss EL Merrill BD Ng KM Higginbottom SK Casavant EP. Transient osmotic perturbation causes long-term alteration to the gut microbiota. Cell. (2018) 173:1742–54.e17. 10.1016/j.cell.2018.05.008 29906449 PMC6061967

[23] Stecher B Chaffron S Käppeli R Hapfelmeier S Freedrich S Weber TC. Like will to like: abundances of closely related species can predict susceptibility to intestinal colonization by pathogenic and commensal bacteria. PLoS Pathogens. (2010) 6:e1000711. 10.1371/journal.ppat.1000711 20062525 PMC2796170

[24] Drago L Toscano M de grandi R Casini V Pace F. Persisting changes of intestinal microbiota after bowel lavage and colonoscopy. Eur J Gastroenterol Hepatol. (2016) 28:532–7. 10.1097/MEG.0000000000000581 27015015

[25] Rizzatti G Lopetuso L Gibiino G Binda C Gasbarrini A. Proteobacteria: a common factor in human diseases. Bio Med Res Int. (2017) 2017:9351507. 10.1155/2017/9351507 29230419 PMC5688358

[26] Shin NR Whon T Bae JW. Proteobacteria: microbial signature of dysbiosis in gut microbiota. Trends Biotechnol. (2015) 33:496–503. 10.1016/j.tibtech.2015.06.011 26210164

[27] Maldonado-Gómez MX Martínez I Bottacini F O'Callaghan A Ventura M van Sinderen D. Stable engraftment of Bifidobacterium longum AH1206 in the human gut depends on individualized features of the resident microbiome. Cell Host Microbe. (2016) 20:515–26. 10.1016/j.chom.2016.09.001 27693307

[28] Boto L. Horizontal gene transfer in evolution: facts and challenges. Proc R Soc B Biol Sci. (2010) 277:819–27. 10.1098/rspb.2009.1679 19864285 PMC2842723

[29] Kodaman N Pazos A Schneider BG Piazuelo MB Mera R Sobota RS. Human and *Helicobacter pylori* coevolution shapes the risk of gastric disease. Proc Natl Acad Sci USA. (2014) 111:1455–60. 10.1073/pnas.1318093111 24474772 PMC3910595

[30] Tyakht AV Alexeev DG Popenko AS Kostryukova ES Govorun VM. Rural and urban microbiota: to be or not to be? Gut microbes. (2014) 5:351–6. 10.4161/gmic.28685 24691073 PMC4153773

[31] Kump P Wurm P Gröchenig HP Wenzl H Petritsch W Halwachs B. The taxonomic composition of the donor intestinal microbiota is a major factor influencing the efficacy of faecal microbiota transplantation in therapy refractory ulcerative colitis. Aliment Pharmacol Ther. (2018) 47:67–77. 10.1111/apt.14387 29052237 PMC5765501

[32] Zuo T Wong SH Lam K Lui R Cheung K Tang W. Bacteriophage transfer during faecal microbiota transplantation in *Clostridium difficile* infection is associated with treatment outcome. Gut. (2018) 67:634–43. 10.1136/gutjnl-2017-313952 28539351 PMC5868238

[33] Mizuno S Masaoka T Naganuma M Kishimoto T Kitazawa M Kurokawa S. Bifidobacterium-rich fecal donor may be a positive predictor for successful fecal microbiota transplantation in patients with irritable bowel syndrome. Digestion. (2017) 96:29–38. 10.1159/000471919 28628918 PMC5637308

[34] Rossen NG Fuentes S van der Spek MJ Tijssen JG Hartman JHA Duflou A. Findings from a randomized controlled trial of fecal transplantation for patients with ulcerative colitis. Gastroenterology. (2015) 149:110–8.e4. 10.1053/j.gastro.2015.03.045 25836986

